# Viral infiltration of pancreatic islets in patients with COVID-19

**DOI:** 10.1038/s41467-021-23886-3

**Published:** 2021-06-10

**Authors:** Charlotte Steenblock, Stefanie Richter, Ilona Berger, Marko Barovic, Janine Schmid, Undine Schubert, Natalia Jarzebska, Anne von Mässenhausen, Andreas Linkermann, Annette Schürmann, Jessica Pablik, Thomas Dienemann, Katja Evert, Roman N. Rodionov, Natalia Y. Semenova, Vsevolod A. Zinserling, Raul R. Gainetdinov, Gustavo Baretton, Dirk Lindemann, Michele Solimena, Barbara Ludwig, Stefan R. Bornstein

**Affiliations:** 1 Department of Internal Medicine III, University Hospital Carl Gustav Carus, Technische Universität Dresden, Dresden, Germany; 2 Institute of Virology, Medical Faculty Carl Gustav Carus, Technische Universität Dresden, Dresden, Germany; 3 CRTD/DFG-Center for Regenerative Therapies, Technische Universität Dresden, Dresden, Germany; 4 Paul Langerhans Institute Dresden (PLID) of the Helmholtz Center Munich at the University Hospital Carl Gustav Carus, Technische Universität Dresden, Dresden, Germany; 5 German Center for Diabetes Research (DZD e. V.), Neuherberg, Germany; 6 University Centre for Vascular Medicine, University Hospital Carl Gustav Carus, Technische Universität Dresden, Dresden, Germany; 7 Department of Anesthesiology and Intensive Care Medicine, University Hospital Carl Gustav Carus, Technische Universität Dresden, Dresden, Germany; 8 Biotechnology Center, Technische Universität Dresden, Dresden, Germany; 9 Department of Experimental Diabetology, German Institute of Human Nutrition Potsdam-Rehbruecke (DIfE), Potsdam, Germany; 10 Institute of Nutritional Science, University of Potsdam, Potsdam, Germany; 11 Department of Pathology, University Hospital Carl Gustav Carus, Technische Universität Dresden, Dresden, Germany; 12 Department of Surgery, University of Regensburg, Regensburg, Germany; 13 Institute of Pathology, University of Regensburg, Regensburg, Germany; 14 S. P. Botkin Clinical Infectious Diseases Hospital, St. Petersburg, Russia; 15 V. A. Almasov Scientific Research Center, St. Petersburg, Russia; 16 Institute of Translational Biomedicine, St. Petersburg State University, St. Petersburg, Russia; 17 St. Petersburg State University Hospital, St. Petersburg State University, St. Petersburg, Russia; 18 Department of Endocrinology and Diabetology, University Hospital Zurich, Zurich, Switzerland; 19 Department of Diabetes, School of Life Course Science and Medicine, King’s College London, London, UK

**Keywords:** Viral infection, Diabetes

## Abstract

Metabolic diseases are associated with an increased risk of severe COVID-19 and conversely, new-onset hyperglycemia and complications of preexisting diabetes have been observed in COVID-19 patients. Here, we performed a comprehensive analysis of pancreatic autopsy tissue from COVID-19 patients using immunofluorescence, immunohistochemistry, RNA scope and electron microscopy and detected SARS-CoV-2 viral infiltration of beta-cells in all patients. Using SARS-CoV-2 pseudoviruses, we confirmed that isolated human islet cells are permissive to infection. In eleven COVID-19 patients, we examined the expression of ACE2, TMPRSS and other receptors and factors, such as DPP4, HMBG1 and NRP1, that might facilitate virus entry. Whereas 70% of the COVID-19 patients expressed ACE2 in the vasculature, only 30% displayed ACE2-expression in beta-cells. Even in the absence of manifest new-onset diabetes, necroptotic cell death, immune cell infiltration and SARS-CoV-2 viral infection of pancreatic beta-cells may contribute to varying degrees of metabolic dysregulation in patients with COVID-19.

## Abstract (web summary)

New-onset hyperglycemia and complications of preexisting diabetes have been observed in COVID-19 patients, however, the underlying mechanisms are not fully understood. Here, the authors show that SARS-CoV-2 is detectable in both endocrine and exocrine cells of the pancreata of patients with COVID-19.

## Introduction

In addition to its known role on the respiratory system, the human coronavirus SARS-CoV-2 has been shown to affect the endocrine system including the pancreas^1–4^. Diabetes and hyperglycemia are associated with an increased risk of severe COVID-19. Furthermore, obesity, hypertension, diabetic nephropathy, and cardiovascular disorders as common comorbidities of diabetes are connected to adverse outcomes of COVID-19^3^. Conversely, new-onset hyperglycemia, ketoacidosis, diabetes, and severe metabolic complications of pre-existing diabetes have been observed in patients suffering from COVID-19^5–11^. Besides single reports of COVID-19 potentially causing or triggering new-onset diabetes^8,10^, this phenomenon is currently analyzed in a newly established systematic registry (http://covidiab.e-dendrite.com/)^11^. It has been shown that human pancreatic alpha and beta-cells derived from pluripotent stem cells may be permissive to SARS-CoV-2 infection^12^, and that SARS-CoV-2 is able to infect and replicate in human cells of the endocrine and exocrine pancreas^13^. Moreover, it has been suggested that coronavirus infiltration in the pancreas may have led to islet damage and acute diabetes in the SARS outbreak in 2002–2003^14^. For COVID-19 patients, a better outcome is observed when a good glycemic control is maintained^15^. Therefore, it has been suggested that ACE2 imbalance in the pancreas causes acute beta-cell dysfunction and a resultant hyperglycemic state^16^. Furthermore, the expression of ACE2 has been shown to be increased upon inflammatory stress suggesting an enhancement of the beta-cell sensitivity to SARS-CoV-2 during inflammatory conditions^17^. Acute pancreatitis following COVID-19 has also been reported^18^.

Still it is a subject of discussion if COVID-19 can indeed cause new-onset diabetes or accelerate pre-existing diabetes or prediabetes^19^. Based on observations that beta-cells do not express virus receptors such as ACE2^20–22^, a direct involvement of virus on beta-cells has been considered unlikely. Therefore, we performed a comprehensive analysis with eleven COVID-19 patients to examine whether islet injury and acute metabolic complications, in the context of an infection with SARS-CoV-2, might be due to direct viral infection of insulin-producing beta-cells. Furthermore, we examined which receptors that might be involved in virus entry.

## Results and discussion

### COVID-19 patient characteristics

To investigate whether SARS-CoV-2 infect pancreatic cells, we examined pancreas autopsies from 20 deceased COVID-19 patients from three different centers in Germany and Russia. As the tissues were from autopsies, they showed varying degrees of autolysis due to prolonged time before autopsy. In the study, we only included tissue, where an easy identification of islets based on H&E stainings (Supplementary Fig. 1) and/or insulin staining was possible. Thereby, 11 patients were selected for further analysis. Six (55%) patients were male, and the patients’ age groups ranged from 40 to 75 years. For ten of the patients, their BMI was known. Two patients exhibited normal weight (BMI 18.5–25 kg/m^2^), four patients were affected by overweight (BMI 25–30 kg/m^2^), and four patients by obesity (BMI > 30 kg/m^2^), two of which had class 3 obesity (BMI > 40 kg/m^2^). One of eleven COVID-19 patients (patient #8) was previously diagnosed with type 2 diabetes (Table 1). As controls, we included autopsy tissues from non-COVID-19 and septic patients. Furthermore, we included tissues from two patients with pancreatitis of one had type 1 diabetes.

**Table 1:** Patient and tissue characteristics.

Patient	Age range	Sex	BMI (kg/m^2^)	Diabetes	COVID-19	Steroid treatment	Autopsy	Blood glucose (admission, mg/dL)	Blood glucose (death, mg/dL)	Blood pH (admission)	Blood pH (death)	Comments
Ctrl #1	–	–	–	–	No	–	No	–	–	–	–	From Zyagen
Ctrl #2	–	–	–	–	No	–	No	–	–	–	–	Tissue from 2011
Ctrl #3	56–60	m	–	No	No	–	Yes	–	–	–	–	Died due to an aortic dissection
Sepsis #1	26–30	m	–	No	No	–	Yes	–	–	–	–	Died due to septic multiorgan failure
Panc. #1	–	–	–	Yes	No	–	No	–	–	–	–	–
Panc. #2	–	–	–	No	No	–	No	–	–	–	–	–
COVID-19 #1	66–70	m	24.7	No	Yes	Yes	Yes	131	162	7.50	7.21	Influenza, RNA quality low
COVID-19 #2	41–45	f	51	–	Yes	No	Yes	–	–	–	–	Died at admission, RNA quality low
COVID-19 #3	66–70	f	35.4	No	Yes	No	Yes	128	127	7.47	7.20	–
COVID-19 #4	71–75	m	26.2	No	Yes	Yes	Yes	130	87	7.36	7.14	RNA quality low
COVID-19 #5	41–45	f	40.4	No	Yes	Yes	Yes	108	268	7.43	7.15	–
COVID-19 #6	61–65	f	20.8	No	Yes	No	Yes	137	146	7.41	7.3	–
COVID-19 #7	51–55	m	29.2	No	Yes	Yes	Yes	126	202	7.48	7.26	–
COVID-19 #8	66–70	m	34	Yes	Yes	Yes	Yes	171	231	7.41	7.29	–
COVID-19 #9	61–65	m	27.8	No	Yes	No	Yes	156	104	7.40	7.39	–
COVID-19 #10	61–65	f	27.7	No	Yes	Yes	Yes	116	86	7.36	7.31	RNA quality low
COVID-19 #11	61–65	m	–	No	Yes	No	Yes	250	91	–	–	Died after 5 days in hospital,RNA quality low

“–” indicates not known.

*m* male, f female, *BMI* body mass index.

In all COVID-19 patients without prediagnosed diabetes, due to our inclusion criteria, intact islets were recognizable. However, a small percentage of impaired islets were also detectable. This damage was noticed at varying degrees, and also in patients without previously diagnosed diabetes (Supplementary Fig. 2). In three COVID-19 patients (#5, #7, and #8), an increase in their blood glucose to more than 200 mg/dL from admission to shortly before they died was observed (Table 1). These three patients were treated with steroids, which are known to induce hyperglycemia in patients already exhibiting a risk of developing diabetes, such as a family history of diabetes, increased age, and obesity^23^. COVID-19 patient #8 was already diagnosed with type 2 diabetes and #5 had class 3 obesity, meaning that in these two cases, the observed hyperglycemia may at least partly be due by the glucocorticoid treatment. However, COVID-19 patient #7 and an additional patient (#1), also treated with steroids, who showed an increase in his arterial blood glucose to 162 mg/dL, were not at an increased risk of developing diabetes before their infection with SARS-CoV-2. This might implicate that the infection have led to hyperglycemia in these patients.

In a recent study, the blood glucose in patients with COVID-19 was investigated, and it was found that in those patients having blood glucose levels >7.0 mmol/L (~125 mg/dL) at admission the mortality rate was the highest^24^. This was the case for eight of our patients. In addition to hyperglycemia, a number of patients (#1, #3, #4, and #5) developed signs of ketoacidosis with arterial blood pH values decreasing to below 7.25 from admission to shortly before death (Table 1).

### SARS-CoV-2 infects human pancreatic cells

**Fig. 1:**
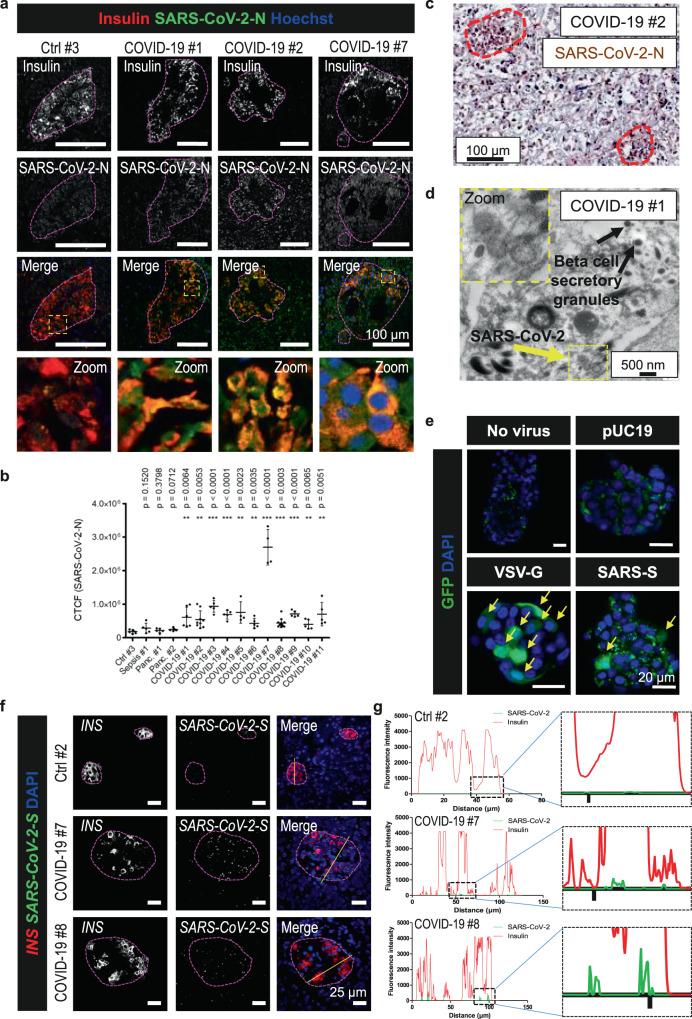
SARS-CoV-2 infection leads to viral infiltration in the pancreas. **a** Immunostainings of pancreas sections from control and COVID-19 patients using antibodies against insulin and SARS-CoV-2-N (sc65653). Representative images from three independent experiments are shown (*n* = 3). Islets are indicated. Scale bars, 100 µm. **b** Dot blots of SARS-CoV-2 cellular fluorescence in beta-cells measured on one (*n* = 1) of three independent stainings in **a**. Quantification of calculated total cellular fluorescence (CTCF) was performed on *n* ≥ 5 cells from *n* ≥ 2 islets per patient. Data were analyzed by unpaired two-sided *t*-test by comparison with Ctrl #3. Data are presented as mean ± SD. *p* values are indicated. **p < 0.01; ***p < 0.001. **c** Immunohistochemistry of pancreas tissue from patient #2 using a different SARS-CoV-2-N antibody (GTX135361) (*n* = 1). Islets are indicated. Scale bar, 100 µm. **d** Electron microscopy image of pancreas tissue from patient #1 showing virus-like particles. Two independent experiments with similar results were performed. Scale bar, 500 nm. **e** Representative images of isolated human islets infected with VSV-G or SARS-CoV-2 pseudoviruses expressing GFP. Results shown are from one of two independent biological experiments (*n* = 2); each with three technical replicates (*n* = 3). pUC19 empty vector. Scale bars, 20 µm. Infected cells are indicated with yellow arrows. **f** Representative images of RNA fluorescence in situ hybridization performed using the RNA scope platform with probes targeting *INS* and *SARS-CoV-2-S* (*n* = 2 independent experiments). Probes against the bacterial *dapB* gene were used as negative control. Islets are indicated. Scale bars, 25 µm. **g** Morphometric analysis of *SARS-CoV-2-S* and *INS* RNA in situ hybridizations. The fluorescence intensities in the different fluorescence channels along the yellow line were measured.

Before performing immunohistochemistry and immunofluorescence stainings for viral antigens, we tested and validated different commercial antibodies recognizing either the SARS-CoV-2 spike (SARS-CoV-2-S) or nucleocapsid (SARS-CoV-2-N) protein (Supplementary Table 1 and Supplementary Fig. 3). Anti-SARS-CoV-2-N from Santa Cruz Biotechnology (sc65653) worked in immunofluorescence. In adrenal negative control tissue (non-COVID-19), there was no signal (Supplementary Fig. 3B), whereas in lung tissue from COVID-19 patient #2, many infected cells were detected with some background fluorescence (Supplementary Fig. 3E). In the pancreas, the antibody exhibited a background fluorescence signal as well but a clearly positive signal was also displayed in tissues from COVID-19 patients (Supplementary Fig. 3F). Therefore, this antibody (sc65653) was used for all immunofluorescence stainings. An antibody against SARS-CoV-2-S (Genetex, GTX135360) did not function in our tests and was not further used. By staining of SARS-CoV-2-N (using sc65653), viral antigens were detected in the endocrine and exocrine pancreata of all the deceased patients, whereas no viral antigens were detected in the negative controls (Fig. 1a, Supplementary Fig. 4). This shows that the infection is systemic and not specific to beta-cells. An analysis of the cellular fluorescence in beta-cells confirmed that in COVID-19 patients the fluorescence level for SARS-CoV-2-N was significantly higher than the background fluorescence observed in control patients. The highest level was observed in patient #7 (Fig. 1b, Table 2).

**Table 2:** Summary of results.

	COVID-19 #1	COVID-19 #2	COVID-19 #3	COVID-19 #4	COVID-19 #5	COVID-19 #6	COVID-19 #7	COVID-19 #8	COVID-19 #9	COVID-19 #10	COVID-19 #11	Ctrl #1	Ctrl #3	Sepsis #1	Panc. #1	Panc. #2	Lung COVID-19 #2	Human islets
Insulin	+++	+++	+++	+++	+++	+++	+++	+++	+++	+++	+++	+++	+++	+++	+++	+++	−	+++
SARS-CoV-2 (beta-cells)	++	++	++	++	++	+	+++	+	++	+	++	−	−	−	−	−	+++(*)	−
ACE2 (beta-cells)	+++	+++	−	−	−	+	−	−	−	+	−	+	−	−	−	+	− (*)	+
ACE2 (endothelial cells)	+	−	+++	+++	+++	+	+++	+++	−	++	+++	++	+++	+++	+++	−	+++	−
pMLKL	+	+++	+	+	+	++	++	++	+	+	nd	−	−	−	+	−	nd	nd

Based on quantifications of fluorescence intensities, the amounts of SARS-CoV-2, ACE2, and pMLKL were estimated. +++ indicates high amount of protein/RNA detected, ++ indicates middle amount detected, + indicates low amount detected, − indicates no protein/RNA detected, nd indicates not determined, (*) indicates alveolar cells.

To confirm the immunofluorescence stainings of viral antigens, we used another antibody (GTX135361) against SARS-CoV-2-N for immunohistochemistry of tissue from COVID-19 patient #2. Similar to the immunofluorescence stainings, we observed a high number of cells positive for viral antigens in both the islets and in the exocrine tissue (Fig. 1c). For COVID-19 patient #1, the paraffin-embedded tissue was re-embedded to perform electron microscopy. After re-embedding, the tissue was partly destroyed. Nevertheless, we observed virus-like particles in cells containing insulin secretory granules (Fig. 1d).

### Human pancreatic islets are permissive to infection with SARS-CoV-2

To further examine if human pancreatic islets are susceptible to infection with SARS-CoV-2, we established a protocol for transduction of isolated human islets with lentiviral vector pseudotypes expressing GFP and containing a truncated SARS-CoV-2 spike (S) protein on their surface (SARS-CoV-2 pseudovirus). Three days after infection, we fixed the intact islets and stained for DAPI (Fig. 1e). In untransduced islets and islets transduced with pseudoviruses containing the empty lentiviral vector just expressing GFP (pUC19), no green cells were detected. As positive control for the methodology, islets were transduced with lentiviral VSV-G pseudotypes (VSV-G pseudovirus). These VSV-G pseudoviruses express the VSV glycoprotein, which binds to the LDL receptor expressed on almost all cell types. Thereby they exhibit a very broad tropism. This is confirmed in our experiment, where around 30% of cells in the islets got infected (Fig. 1e). On the other hand, SARS-CoV-2 pseudoviruses has a much lower tropism as they will only infect cells expressing ACE2 and the co-factor TMPRSS2. In islets transduced with SARS-CoV-2 pseudoviruses, 1–3 cells per islet got infected showing that islet cells are susceptible to infection with SARS-CoV-2 (Fig. 1e). These results are consistent with our unpublished results using SARS-CoV-2 pseudovirus transduction of HEK293 cells overexpressing ACE2, where we see around 15% transduction.

### SARS-CoV-2 viral RNA is detectable in islets

By using RNA in situ hybridization we detected viral SARS-CoV-2 RNA in islets in six of the eleven COVID-19 patients (#3 and #5-9) (Fig. 1f, Supplementary Fig. 5 and Table 2). An archival control (Ctrl #2, pancreatic tissue collected in 2011) was used to ensure specificity of SARS-CoV-2 probes. The *SARS-CoV-2-S* signal was very low compared to the *INS* signal, but morphometric analysis showed that the viral RNA was detected in the insulin-producing beta-cells (Fig. 1g). In the exocrine tissue, no viral RNA was detected. In other five of the eleven patient tissues (#1-2, #4, #10, and #11), RNA quality was low because of tissue autolysis and neither *INS* nor *SARS-CoV-2-S* was detectable. The reason why only small amounts of viral RNA were detected (Fig. 1g) might be due to the fact that all patients, except for #2 and #11, died more than 3 weeks after the initial infection with SARS-CoV-2. This could mean that no or just few active virus particles were still present.

### ACE2 and TMPRSS2 are expressed in endocrine cells

Angiotensin-converting enzyme 2 (ACE2) and the protease TMPRSS2 are known to be important for SARS-CoV-2 entry^25^. The expression of ACE2 in the pancreas is a matter of debate and contradictory results have been reported. For example, three different studies showed a higher expression of ACE2 in the exocrine duct cells than in the islets^20–22^, whereas other studies showed that ACE2 is preferentially expressed in beta-cells^13,17^. Expression of ACE2 was detected in the microvasculature in both the exocrine and endocrine pancreas^17,20,21^. Our own gene expression data on human islets and EndoC1 beta-cells shows a very low expression of ACE2 in beta-cells (unpublished). By single-cell sequencing, we have shown that in pancreata from mice, both ACE2 and an alternative entry receptor, dipeptidyl peptidase-4 (DPP4 also known as CD26) are highly expressed in delta-cells followed by beta- and alpha-cells (unpublished). To clear these contradictory results, we have examined the expression of ACE2 and TMPRSS2 in pancreatic tissue from eleven patients that died of COVID-19 and compared to control tissues. First, we tested and validated different antibodies against these potential receptors and proteases involved in virus entry. We selected three commercially available ACE2 antibodies and two TMPRSS2 antibodies. These antibodies were tested on paraffin and cryosections from human pancreas or adrenal tissue (Supplementary Table 1 and Supplementary Fig. 3). For paraffin sections, we performed antigen retrieval by boiling for 3 min in citrate buffer in an NxGen pressure cooker. Two of the ACE2 antibodies (Thermo Fisher, MA531394 and Abcam, ab15348) showed similar stainings (Supplementary Fig. 3A, D) and both were used throughout the study. A third ACE2 antibody, which has been widely used, is AF933 from R&D Systems. This antibody did not work under the conditions we tested and was not further used (Supplementary Fig. 3B). A TMPRSS2-antibody from Abcam (ab92323) worked nicely (Supplementary Fig. 3A). Anti-TMPRSS2 from Santa Cruz Biotechnology (sc515727) showed a similar staining pattern but also in the nucleus (Supplementary Fig. 3C). Therefore, ab92323 was chosen for further immunofluorescence stainings.

**Fig. 2:**
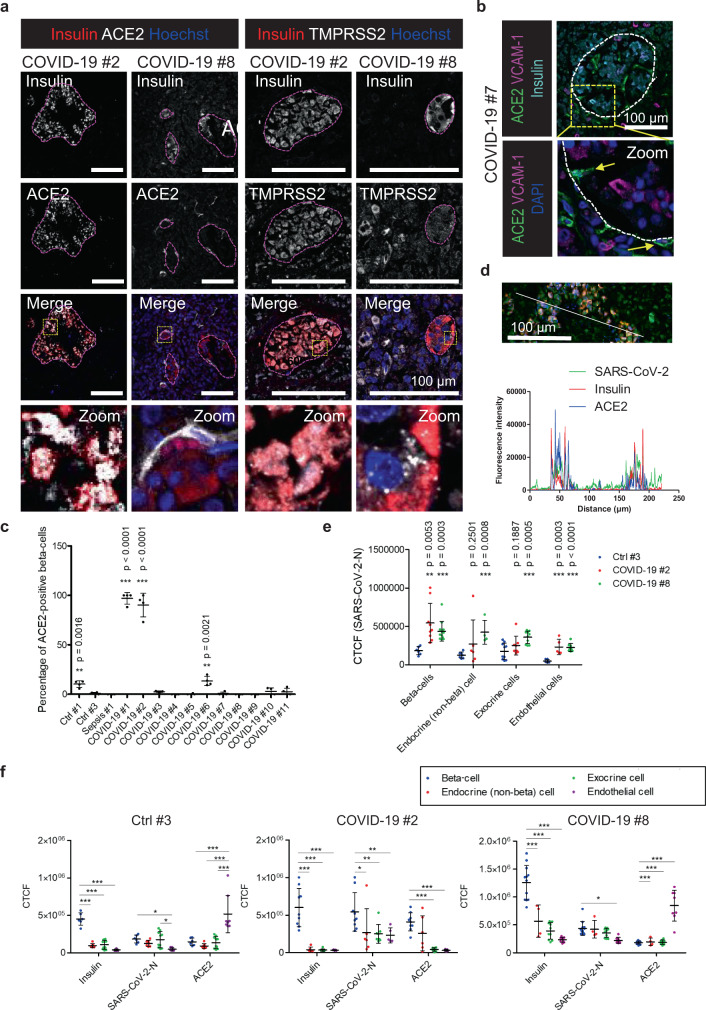
SARS-CoV-2 receptors are expressed in the human pancreas. **a** Pancreas sections from COVID-19 patients were immunostained for insulin to mark beta-cells. Additionally, double stainings for ACE2 and TMPRSS2 were performed. Representative images from three independent experiments (*n* = 3) are shown. Islets are indicated. Scale bars, 100 µm. **b** Triple immunostaining for ACE2, insulin and the endothelial marker VCAM-1. Representative image from two independent experiments (*n* = 2) are shown. Islets are indicated. Scale bar, 100 µm. **c** Dot blot showing the percentage of beta-cells positive for ACE2 in control and COVID-19 patients. All insulin-positive cells in *n* ≥ 3 islets per patient were counted from three independent experiments (*n* = 3). Data were analyzed by unpaired two-sided *t*-test by comparison with Ctrl #3. Data are presented as mean ± SD. **d** Morphometric analysis of an ACE2/Insulin/SARS-CoV-2-N staining of pancreatic tissue from COVID-19 patient #2 (*n* = 1). The fluorescence intensities in the indicated fluorescence channels along the white line were measured. Scale bar, 100 µm. **e** Calculated total cellular fluorescence (CTCF) of SARS-CoV-2 in insulin-positive and -negative islet cells, exocrine cells, and endothelial cells. Data were analyzed by unpaired two-sided *t*-test by comparison with Ctrl #3. Data are presented as mean ± SD. **f** CTCF of ACE2 and SARS-CoV-2 was calculated in insulin-positive and -negative islet cells, exocrine cells and endothelial cells. Data were analyzed by two-way ANOVA and Bonferroni posttest. Data are presented as mean ± SD. *p < 0.05; **p < 0.01; ***p < 0.001. In **e** and **f**, quantification of CTCF was calculated based on *n* ≥ 5 cells from *n* ≥ 2 islets per patient from one (*n* = 1) out of three independent experiments.

Two different types of ACE2 expression was detected in the pancreatic islets (Fig. 2a and supplementary Fig. 6). As observed in other studies^17,20,21^, in nine COVID-19 patients and one control tissue (Ctrl #3), ACE2 was expressed in the microvasculature (Fig. 2a, Supplementary Fig. 6 and Table 2). This was identified by double staining for the endothelial marker VCAM-1 (Fig. 2b, Supplementary Fig. 7), which is known to be upregulated in endothelial cells after inflammation leading to lymphocyte infiltration^26^. The same pattern we observed in tissue from septic patients and in non-COVID-19 patients with pancreatitis. Conversely, in one control (Ctrl #1) and three patients (COVID-19 #1, #2, and #6), ACE2 was highly expressed in beta-cells (Fig. 2a and supplementary Fig. 6) fitting with the study from Fignani et al., showing expression of ACE2 in beta-cells and that this expression was increased in response to pro-inflammatory cytokines^17^. In COVID-19 patients #1 and #2, more than 90% of the insulin-producing beta-cells were positive for ACE2. In Ctrl #1 and COVID-19 patient #6, around 10% of the beta-cells expressed ACE2, whereas all other patients expressed ACE2 in less than 3% of the beta-cells (Fig. 2c). For COVID-19 patient #2, morphometric analysis showed that SARS-CoV-2 viral antigens were found both in the ACE2-positive beta-cells and in ACE2-negative exocrine cells (Fig. 2d). Analysis of the cellular fluorescence density further confirmed that in the two ACE2-types of patients, independent of their ACE2 expression, SARS-CoV-2 viral antigens were detected in both endothelial, exocrine and endocrine cells (beta-cells and non beta-cells) (Fig. 2e). The highest SARS-CoV-2 level was detected in beta-cells of COVID-19 patient # 2 also showing the highest ACE2 level in beta-cells (Fig. 2f). Conversely, patients with a high ACE2 expression in endothelial cells did not show a significantly higher level of SARS-CoV-2 virus particles in these cells (Fig. 2f). However, as nine of the eleven patients died at least 3 weeks after the initial infection, the virus particles in the vasculature might not be detectable anymore. Most other studies related to ACE2 expression are based on non-COVID-19 tissue or on tissue from a few COVID-19 patients, which might explain why they did not observe any expression of ACE2 in the beta-cells^20–22^. It still needs to be clarified if the high expression of ACE2 in beta-cells in a small number of COVID-19 patients was due to the inflammation response in relation to the infection with SARS-CoV-2 as suggested by Fignani et al.^17^, or if they were infected because they already had a high ACE2 expression. It has previously been shown that the expression of ACE2 is sex- and age-specific^27,28^. However, in our study with 17 donors in total, we do not see any tendencies in this direction.

 In the COVID-19 autopsy material, the expression varied between the patients explaining why previous studies have shown very different results in relation to expression of ACE2 in the pancreas.

In one of our control tissues (Ctrl #1), TMPRSS2 was highly expressed in the islets, whereas no expression was observed in the exocrine pancreas. However, in all other non-COVID-19 and COVID-19 tissues tested, TMPRSS2 was expressed in both endocrine and exocrine tissue (Fig. 2a and Supplementary Fig. 8).

### Alternative SARS-CoV-2 entry factors are expressed in beta-cells

**Fig. 3:**
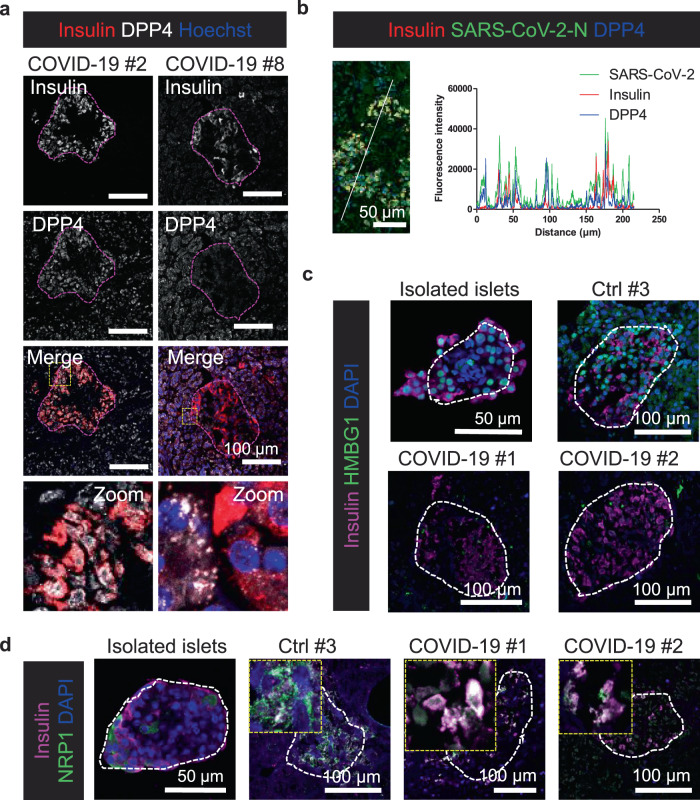
Alternative receptors and factors potentially facilitating virus entry. **a** Pancreas sections from control patients and deceased COVID-19 patients were immunostained for insulin to mark beta-cells. Additionally, double stainings for DPP4 were carried out. Representative images from two independent experiments (*n* = 2) are shown. Islets are indicated. Scale bars, 100 µm. **b** Morphometric analysis of a DPP4/Insulin/SARS-CoV-2-N staining of pancreatic tissue from COVID-19 patient #2 (*n* = 1). The fluorescence intensities in the indicated fluorescence channels along the white line were measured. Scale bar, 50 µm. **c** Double stainings for insulin and HMBG1. Representative images from two independent experiments (*n* = 2) are shown. Islets are indicated. Scale bars, 50 µm for isolated islets, otherwise 100 µm. **d** Double stainings for insulin and NRP1. Representative images from two independent experiments (*n* = 2) are shown. Islets are indicated. Scale bars, 50 µm for isolated islets, otherwise 100 µm.

As not just ACE2-positive cells were positive for viral particles in the pancreas, we decided to investigate the expression of alternative receptors and factors shown to facilitate virus entry. The MERS-CoV receptor DPP4 has been suggested as an alternative receptor for SARS-CoV-2^29^, and has has previously been shown to be expressed by cells of the immune system as well as on epithelia and endothelia cells in pancreas, lungs, and kidney^30^. In the non-COVID-19 and COVID-19 patients in our study, DPP4 was expressed in both the endocrine and exocrine pancreas (Fig. 3a and Supplementary Fig. 9). For COVID-19 patient #2, morphometric analysis showed that viral antigens were found in DPP4-positive beta-cells in both the endocrine and exocrine pancreas (Fig. 3b) suggesting that DPP4 could at least partly be responsible for virus entry in the ACE2-negative beta-cells in this patient. Two other proteins, HMBG1 and NRP1 were recently shown to be involved in SARS-CoV-2 virus entry^31,32^. In human islets and in control patient tissue, HMBG1 was expressed in most cells but surprisingly, in the islets from the COVID-19 patients only a few cells positive for HMBG1 were detected (Fig. 3d). This suggests that HMBG1 is downregulated after infection with SARS-CoV-2, also explaining the islet impairment, as HMBG1 has previously been shown to play an important role in inflammation by inhibiting acute pancreatitis^33^. NRP1, shown to facilitate virus entry^31^, was detected in both isolated, control and COVID-19 islets (Fig. 3d) suggesting that in beta-cells with a low ACE2 expression, virus uptake could be facilitated by NRP1, or alternatively DPP4 could work directly as a receptor. Due to their insulin-secreting capacity beta-cells have a high protein turnover, which might explain why these cells are susceptible to infection with SARS-CoV-2 despite a low expression of ACE2. Thereby, beta-cells are useful targets for the viruses, which hijack these cells for their own replication. Similar results have been observed with enteroviruses, which have been suggested to cause diabetes^34^. We have also previously shown that a virus-like infection of human islets with polyI:C leads to a decrease in insulin-production in beta-cells^35^.

Both ACE2 and DPP4 are established transducers of metabolic signals and pathways regulating inflammation, renal and cardiovascular physiology, and glucose homeostasis^36^. DPP4 inhibitors are widely used for the treatment of type 2 diabetes^37^, but until now there is no clinical evidence that drugs targeting ACE2- or DPP4-related pathways show any benefits or harm in relation to human coronavirus infections^36^.

### SARS-CoV-2 infection is associated with necroptosis in endocrine and exocrine cells

**Fig. 4:**
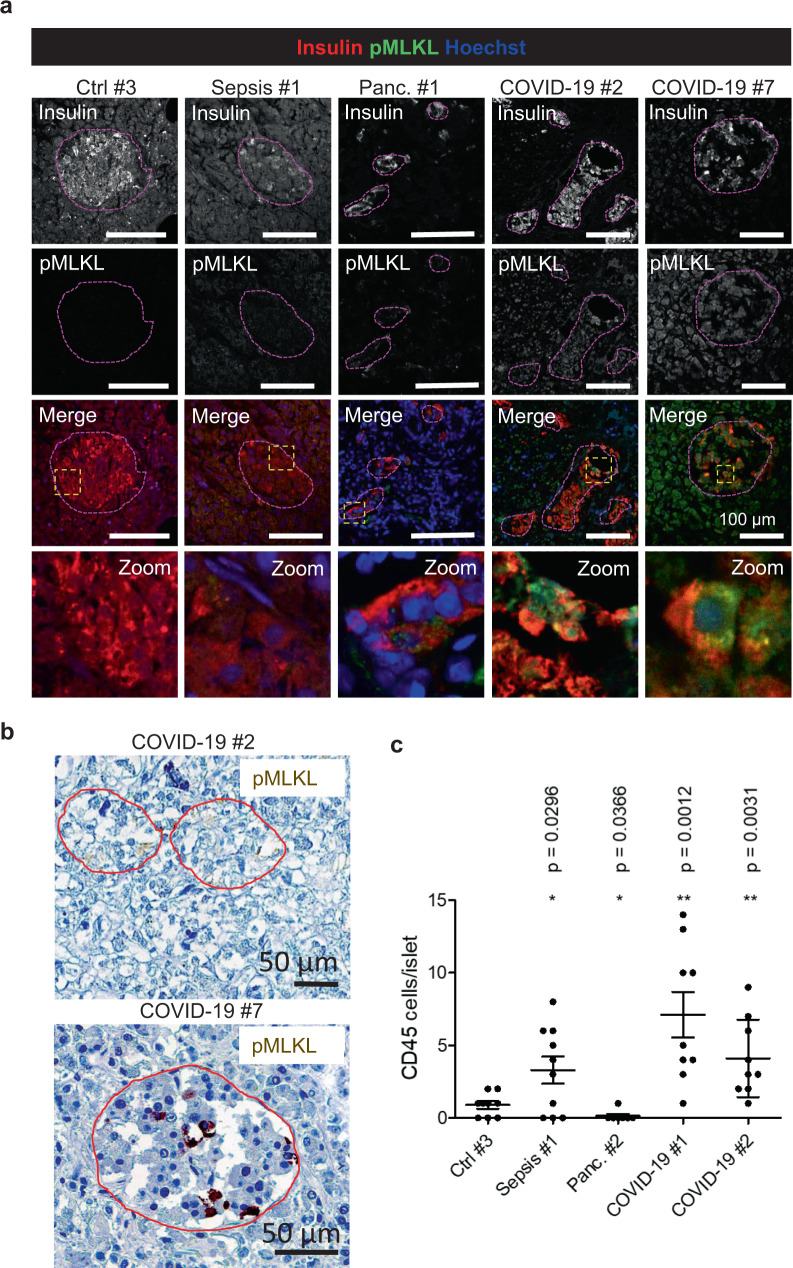
SARS-CoV-2 infection leads to necroptosis and immune cell infiltration in the pancreas. **a** Immunostainings of pancreas sections from non-COVID-19 patients (control, septic, and with pancreatitis) and from COVID-19 patients using antibodies against insulin and pMLKL marking necroptotic cells. Representative images from three independent experiments (*n* = 3) are shown. Scale bars, 100 µm. **b** Immunohistochemistry of pancreas tissue from patient #2 and #7 marking pMLKL-positive cells (*n* = 1). Islets are indicated. Scale bars, 50 µm. **c** On immunostainings of pancreas sections from non-COVID-19 patients and from COVID-19 patients stained against insulin and CD45 marking hematopoietic cells, the amount of CD45-positive cells per islet were quantified in *n* ≥ 7 islets per patient from two independent experiments (*n* = 2). Data were analyzed by unpaired two-sided *t*-test by comparison with Ctrl #3. Data in dot plot are presented as mean ± SD. *p*-values are indicated. *p < 0.05; **p < 0.01.

The innate and the adaptive immune systems control surveillance of virally infected cells and remove these by means of apoptosis or necroptosis. While many regulated and nonregulated pathways of necrosis exist, necroptosis is following an evolutionarily conserved pattern, mediated by kinases such as RIPK1 and RIPK3. Importantly, the hallmark feature of necroptosis is a phosphorylation-site in the pseudokinase mixed lineage kinase domain like (MLKL) protein^38^. pMLKL may then oligomerize and cause the loss of plasma membrane integrity^39^. This process is opposed by the ESCRT-III membrane repair complex, which may be hijacked by viruses^40^. Human coronavirus infection has been suggested to trigger necroptosis^41^ and given the necrotic morphology of the islets of the COVID-19 patients, we hypothesized that SARS-CoV-2-triggered necroptosis might be the cause. To investigate the role of necroptosis in the pancreata, we performed immunohistochemistry and immunofluorescence staining for pMLKL. First, we validated the pMLKL-antibody by inducing necroptosis in HT29 cells^42^. This showed that pMLKL was specific for necroptotic cells (Supplementary Fig. 3G). pMLKL-positive cells were detected in all COVID-19 patient samples (Fig. 4a, b and Supplementary Figs. 10 and 11A). However, less than 10 islets per patient were highly positive for pMLKL (Supplementary Fig. 11B) implicating why just a small percentage of islets were impaired.

Necroptosis requires ATP and is therefore unlikely to happen postmortem^43^ supported by the fact that control autopsy tissue remained pMLKL-negative (Fig. 4a and Supplementary Figs. 10 and 11A). This suggests that SARS-CoV-2 viral infiltration leads to pMLKL positivity, most likely associated with the necroptosis signaling pathway^44^, and might explain why new-onset diabetes has been reported after infection with SARS-CoV-2^6,8,10^. In non-COVID-19 tissue from patients with pancreatitis, pMLKL-positivity was observed, whereas in autopsy tissue from septic patients no pMLKL was detectable ruling out general immune-mediated damage (Supplementary Fig. 11A). The clinical data, we received were limited but it appears that patients with elevated glucose levels presented a significant viral load in beta-cells, a high expression of ACE2 either in beta- or endothelial cells and that necroptosis was induced. Especially, for COVID-19 patients #1 and #7, there might be a correlation between viral load, islet impairment, hyperglycemia, and necroptosis (Table 2). Patient #8 was already diagnosed with diabetes before his infection with SARS-CoV-2, whereas for patient #2 who died immediately after admission, we unfortunately do not have any clinical data. However, as she had class 3 obesity, she was at high risk for obesity associated hyperglycemia and diabetes.

### Islets infected with SARS-CoV-2 are infiltrated with immune cells

To assess whether pancreata from COVID-19 patients were infiltrated with immune cells, we examined the amount of CD45-positive cells in the tissues. Compared to control autopsy tissue, the amount of CD45-positive cells in the islets of COVID-19 and septic patients increased significantly (Fig. 4c and Supplementary Fig. 12A). In the exocrine tissue, immune cells were detected in the COVID-19 patients as well, and in the septic control tissue, there was a massive infiltration in the exocrine pancreas (Supplementary Fig. 12A). This suggests that in COVID-19 patients, although both exocrine tissue and endocrine tissues are infiltrated, in some patients mainly the islets are affected, which also fits with the fact that in these patients we observed the highest virus loads in the beta-cells (Fig. 2e, f). The tissue surrounding the pancreas was also infiltrated with lymphocytes (Supplementary Fig. 12B). These data implicate that beta-cell infection with SARS-CoV-2 might lead to either direct or indirect impairment of the beta-cells causing variable degrees of metabolic dysregulation.

### Comorbidities may increase the risk of severe COVID-19

Recently, it was shown that age alone does not account for an increased risk of a severe outcome due to COVID-19 infection^45^. This fits with our study, where only four of the eleven patients were older than 65 years of age. In contrast, just two patients (#1 and #6) were within the normal weight range. However, in addition to his infection with SARS-CoV-2, patient #1 was infected with influenza virus (Table 1). Two female patients died before age 45 (patients #2 and #5), and both had class 3 obesity. Six patients had overweight or obesity with BMI values between 25 and 35 kg/m^2^ (patients #3, #4 and #7-10) and for patient #8 it was known that he had type 2 diabetes. These data support the fact that other comorbidities increase the risk of severe outcomes due to COVID-19.

## Conclusion

In conclusion, using human islets and autopsy tissue from patients that died of COVID-19, we provided clear proof that beta-cells are permissive to infection with SARS-CoV-2. The mechanism of virus entry are not completely clear at this point as ACE2 is only expressed in beta-cells in a subset of patients. Therefore, other receptors/factors may be involved in facilitating uptake of SARS-CoV-2 into beta-cells. We have clearly shown that SARS-CoV-2 may induce a local inflammation and may be associated with necroptotic cell death in islets, but the current study cannot answer in detail the mechanism that may lead to islet impairment and metabolic dysregulation. Further cellular models and animals must be used to address this question^46^. Clinicians need to be aware of the potential of SARS-CoV-2 to lead to direct or indirect impairment of islet function.

## Methods

### Autopsies

Autopsies were conducted on one patient that died due to an aortic dissection, one patient that died because of septic multiorgan failure and on 20 patients who died of COVID-19. Pancreatic tissue was fixed in formalin and embedded in paraffin. The autopsies were performed at Institute of Pathology at Universitätsklinikum Carl Gustav Carus in Dresden, at Institute of Pathology at Universität Regensburg or at S. P. Botkin Clinical Infectious Diseases Hospital in St. Petersburg. Autopsy samples from control and septic patients were obtained from the Institute of Pathology at Universitätsklinikum Carl Gustav Carus in Dresden. Tissue samples were received from the pathology institutes with respective approval of local ethics committees and collected in the frame of the German Registry for COVID-19 Autopsies (DeRegCOVID). Written informed consent to perform autopsies and to use the tissues for research purposes were obtained from patient relatives. From the 20 COVID-19 patients, 11 were selected for further analysis. The remaining tissues were excluded from the study due to the bad tissue quality making it impossible to identify islets based on insulin staining. Normal adrenal and pancreatic tissues were obtained from Zyagen. Clinical information is provided in Table 1

### Immunohistochemistry/immunofluorescence

Paraffin slides were deparaffinized in Neo-Clear (Merck) and rehydrated through a descending graded ethanol series. Antigen retrieval was performed in citrate retrieval buffer pH 6.0, using a Decloaking Chamber NXGEN (Menarini Diagnostics) at 110 °C for 3 min.

For immunofluorescence, sections or infected islets fixated in 4% PFA were blocked in blocking buffer (PBS containing 1% BSA, 0.1% Triton X-100, 5% goat serum for 1 h at room temperature, followed by incubation with primary antibody diluted in PBS containing 1% BSA, 5% goat serum at 4 °C overnight. Slides were washed in PBS and incubated with appropriate fluorophore-conjugated secondary antibodies in PBS for 2 h at room temperature. Slides were washed in PBS. Nuclei were stained with 4′-6-diamidino-2-phenylindole (DAPI; Thermo Fisher Scientific) and slides were mounted with fluorescent mounting medium (Aqua-Poly/Mount; Polysciences).

For immunohistochemistry, deparaffinized sections were blocked and stained using appropriate antibodies and Vectastain ABC Kit Peroxidase and AEC Substrate Kit for Peroxidase following the instructions of the manufacturer (Vector Laboratories). Counterstaining with hematoxylin was performed.

### Confocal laser scanning microscopy and fluorescence microscopy

Confocal imaging was performed with a Zeiss LSM 700 or LSM 880 inverted confocal laser scanning microscope and ZEN 2010 software (Zeiss). Image processing and analysis were carried out using ImageJ version 1.53c software. Cellular fluorescence was measured using ImageJ, where the corrected total cell fluorescence (CTCF) was calculated with the formula: CTCF = Integrated density − (Area of selected cell × Mean fluorescence of background readings).

### RNA in situ hybridization

RNA scope was performed using the RNAscope® Multiplex Fluorescent Reagent Kit v2 on formalin-fixed paraffin-embedded tissue using a probe against SARS-CoV-2-S (V-nCoV2019-S-C3, cat. No. 848561-C3), and a probe against insulin (Hs-INS, cat. No. 313571) following the instructions of the manufacturer (ACDBio). Internal assay positive control (against *POLR2A* and *UBC*) and negative control (bacterial gene *dapB*) were used in addition to the staining of archival pancreatic tissue collected 9 years ago to ensure specificity of the SARS-CoV-2 probe.

### Electron microscopy

Formalin-fixed and paraffin-embedded pancreas tissue from patient #1 was re-embedded for electron microscopy. Briefly, Toluol (Carl Roth) was added to small pieces of tissue embedded in paraffin and incubated at 40 °C while shaking. This step was repeated twice followed by rehydration through a descending graded ethanol series. Afterwards, the samples were incubated in 1% osmium tetroxide (OsO_4_) in pure water for 3 h followed by three times washing in pure water. After dehydration through an ascending graded ethanol series the tissue was infiltrated with resin (EtOH/Epon mixture) mixed 3:1 for 3.5 h. EtOH/Epon mixed 1:1 was then added ON and next day EtOH/Epon mixed 1:3 was added for 3.5 h. Pure Epon was added ON followed by polymerization for 48 h at 60 °C. Ultrathin sections (80 nm) were poststained by incubation in 2% uranyl acetate in pure water for 5 min followed by three times washing in pure water and incubation in 0.4% lead citrate for 2 min. After washing 3 × 3 min in pure water samples were analyzed in a CM 10 electron microscope (Philips).

### Human islet isolation

Human islets of Langerhans were isolated from resected pancreas tissue with appropriate consent and ethical approval at the University Hospital Carl Gustav Carus Dresden according to a modified Ricordi method^47,48^. Briefly, Collagenase, neutral protease (Nordmark), and Pulmozyme (Roche) were infused into the main pancreatic duct. Islets were separated from exocrine tissue by centrifugation on a continuous Biocoll gradient (Biochrom AG) in a COBE 2991 cell processor (Lakewood). Following isolation, islets were cultured in RPMI 1640 supplemented with 5.5 mM glucose, 20 mM Hepes, 10% FBS, 0.1% penicillin/streptomycin.

### Pseudovirus production

Lentiviral VSV-G or SARS-CoV-2-S pseudotypes (pseudovirus) were generated by polyethylenimine-mediated transient transfection of 293 T packaging cells^49,50^. Briefly, packaging cells in 10 cm dishes were co transfected with a lentiviral transfer vector containing a spleen focus forming U3 promoter driven firefly luciferase–eGFP fusion protein reporter cassette (pCL6 Luci-EG wo) and HIV-1 Gag/Pol packaging vector (pCD/NL-BH) and the respective glycoprotein packaging vector encoding VSV-G (pcziVSV-G) or containing an expression-optimized, C-terminally truncated SARS-CoV-2 spike protein ORF (pCG1 hSARS-CoV-2 S∆c18) at a 2:2:1 ratio and 16 µg total DNA. Cell-free virus supernatants were harvested 48 h post transfection and stored in aliquots at −80 °C until further use.

### Transduction of human islets

Islets were transferred to low attachment 96 well plates (Corning) and 3 days after isolation they were transduced with cell-free lentiviral pseudotype vector supernatant diluted 1:2 in RPMI 1640 supplemented with 5.5 mM glucose, 20 mM hepes, 10% FBS, 0.1% penicillin/streptomycin by spinoculation at 270 × *g*, 30 °C for 2 h, followed by incubation at 37 °C, 5% CO_2_ in a tissue culture incubator until fixation in 4% PFA for immunofluorescence analysis at 72 h post infection.

### Statistical analysis

Statistical analysis was performed using GraphPad Prism version 5.01 (GraphPad Software Inc.). Statistical significance was determined using two-way ANOVA followed by a Bonferroni multiple comparison test correction where appropriate. Unpaired two-tailed Student’s *t*-test was performed for comparison of two means. The significance was defined as: not significant (ns) *P* > 0.05; *P < 0.05; **P < 0.01; ***P < 0.001.

### Reporting summary

Further information on research design is available in the Nature Research Reporting Summary linked to this article.

## Supplementary information

Supplementary Information

Peer Review File

Reporting Summary

## Data Availability

The authors declare that the data supporting the findings of this study are available within the paper and its supplementary information files. Source data are provided with this paper.

## Source data

Source data
