# Supplementary material for: Viral infiltration of pancreatic islets in patients with COVID-19
Source: Nat Commun. 2021 Jun 10;12:3534. doi: 10.1038/s41467-021-23886-3 (PMC8192507; doi:10.1038/s41467-021-23886-3)
Supplement: Supplementary file 1 — Supplementary Information [file 41467_2021_23886_MOESM1_ESM.pdf]

Supplementary files, Steenblock et al.

Supplementary Table 1. List of antibodies used

Antibody	SOURCE	Dilution	Comments
Guinea pig polyclonal anti-insulin	Abcam<br>Cat# ab7842	1:100	Works very well for IF.<br>IHC wasn't tested.
Rabbit polyclonal anti-ACE2	Abcam<br>Cat# ab15348	1:500	Works well for IF.<br>IHC wasn't tested.
Mouse monoclonal anti-ACE2 (clone CL4013)	Thermo Fisher Scientific<br>Cat# MA5-31394	1:10,000	Works ok for IF.<br>IHC wasn't tested.
Goat polyclonal anti-ACE2	R&D Systems<br>Cat# AF933	1:100	Doesn't work for IF.<br>IHC wasn't tested.
Rabbit polyclonal anti-DPP4	Proteintech<br>Cat# 10940-1-AP	1:50	Works well for IF.<br>IHC wasn't tested.
Rabbit monoclonal anti-TMPRSS2 (clone EPR3861)	Abcam<br>Cat# ab92323	1:1,000	Works well for IF.<br>IHC wasn't tested.
Mouse monoclonal anti-TMPRSS2 (clone H-4)	Santa Cruz Biotechnology<br>Cat# sc515727	1:100	Seems to work for IF but also nuclear staining.
Rabbit monoclonal anti-pMLKL (clone EPR9514)	Abcam<br>Cat# ab187091	1:250	Works well for IF and IHC.
Mouse monoclonal anti-SARS-CoV-2-N (clone FIPV3-70)	Santa Cruz Biotechnology<br>Cat# sc65653	1:100	Works ok for IF.<br>IHC wasn't tested.
Rabbit polyclonal anti-SARS-CoV-2-N	Genetex<br>Cat# GTX135361	1:500	Works ok for IHC.<br>IF wasn't tested.
Rabbit polyclonal anti-SARS-CoV-2-S	Genetex<br>Cat# GTX135360	1:500	Didn't work for IF.<br>IHC wasn't tested.
Rabbit monoclonal anti-VCAM-1 (clone EPR5047)	Abcam<br>Cat# ab134047	1:250	Works well for IF.<br>IHC wasn't tested.
Rabbit monoclonal anti-NRP1 (clone EPR3113)	Abcam<br>Cat# ab81321	1:250	Works well for IF.<br>IHC wasn't tested.
Rabbit monoclonal anti-HMBG1 (clone EPR3507)	Abcam<br>Cat# ab79823	1:250	Works well for IF.<br>IHC wasn't tested.
Rabbit monoclonal anti-CD45 (clone D9M8I)	Cell Signaling<br>Cat# 13917	1:200	Works well for IF.<br>IHC wasn't tested.
A488-goat-anti-rabbit	Jackson ImmunoResearch<br>Cat# 111-545-144	1:500	-
Cy3-goat-anti-rabbit	Jackson ImmunoResearch<br>Cat#111-165-144	1:500	-
A647-goat-anti-rabbit	Thermo Fisher Scientific<br>Cat# A21246	1:200	-
Cy3-donkey-anti-guinea pig	Jackson ImmunoResearch<br>Cat# 706-165-148	1:500	-
A488-goat-anti-mouse	Abcam<br>Cat# ab150113	1:500	-
Cy3-donkey-anti-goat	Jackson ImmunoResearch<br>Cat# 705-165-147	1:500	-

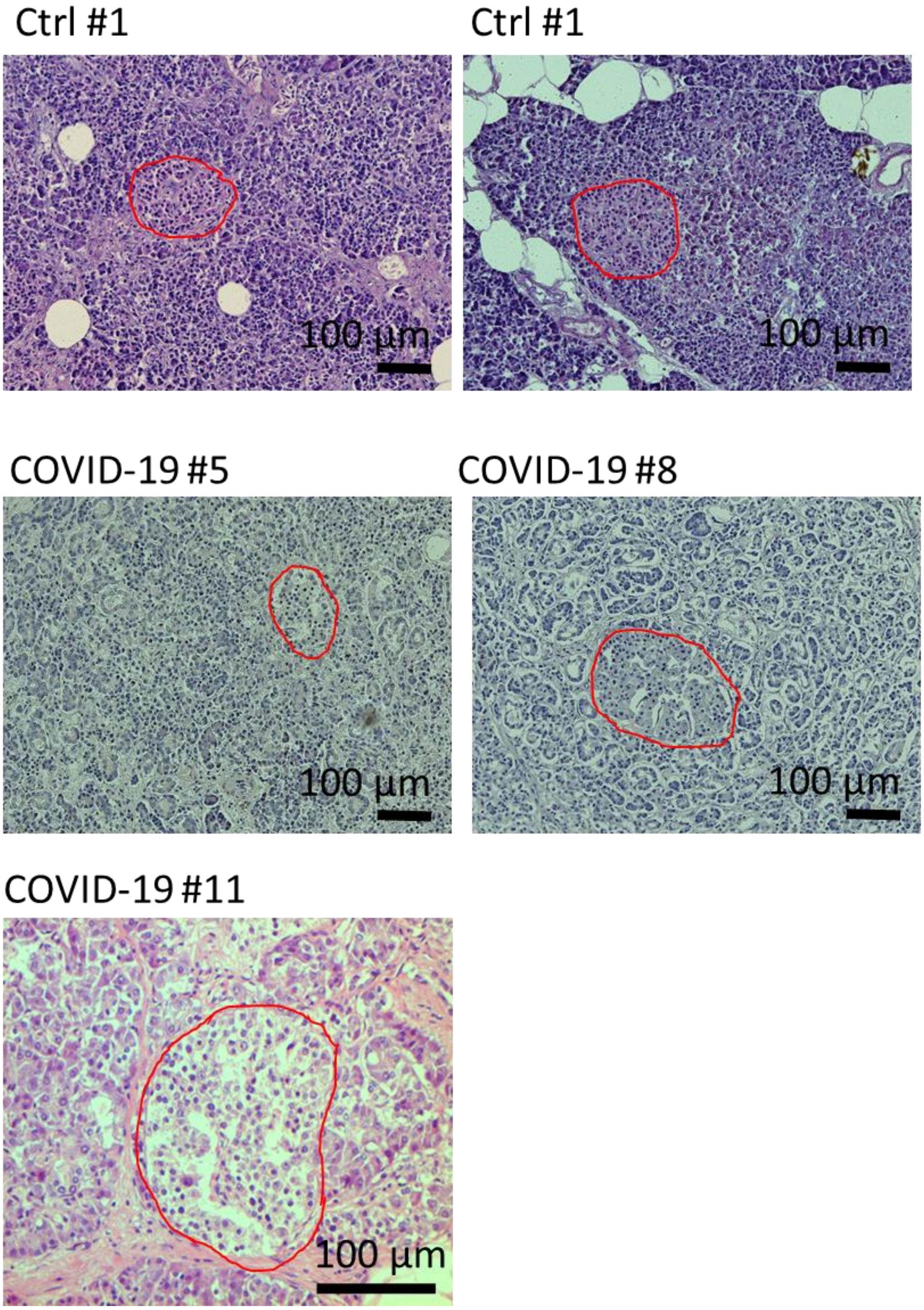

Supplementary Figure 1. H&E stainings of selected pancreas control and COVID-19 patient tissues. Representative images from one experiment (n=1) are shown. Scale bars, 100 µm.

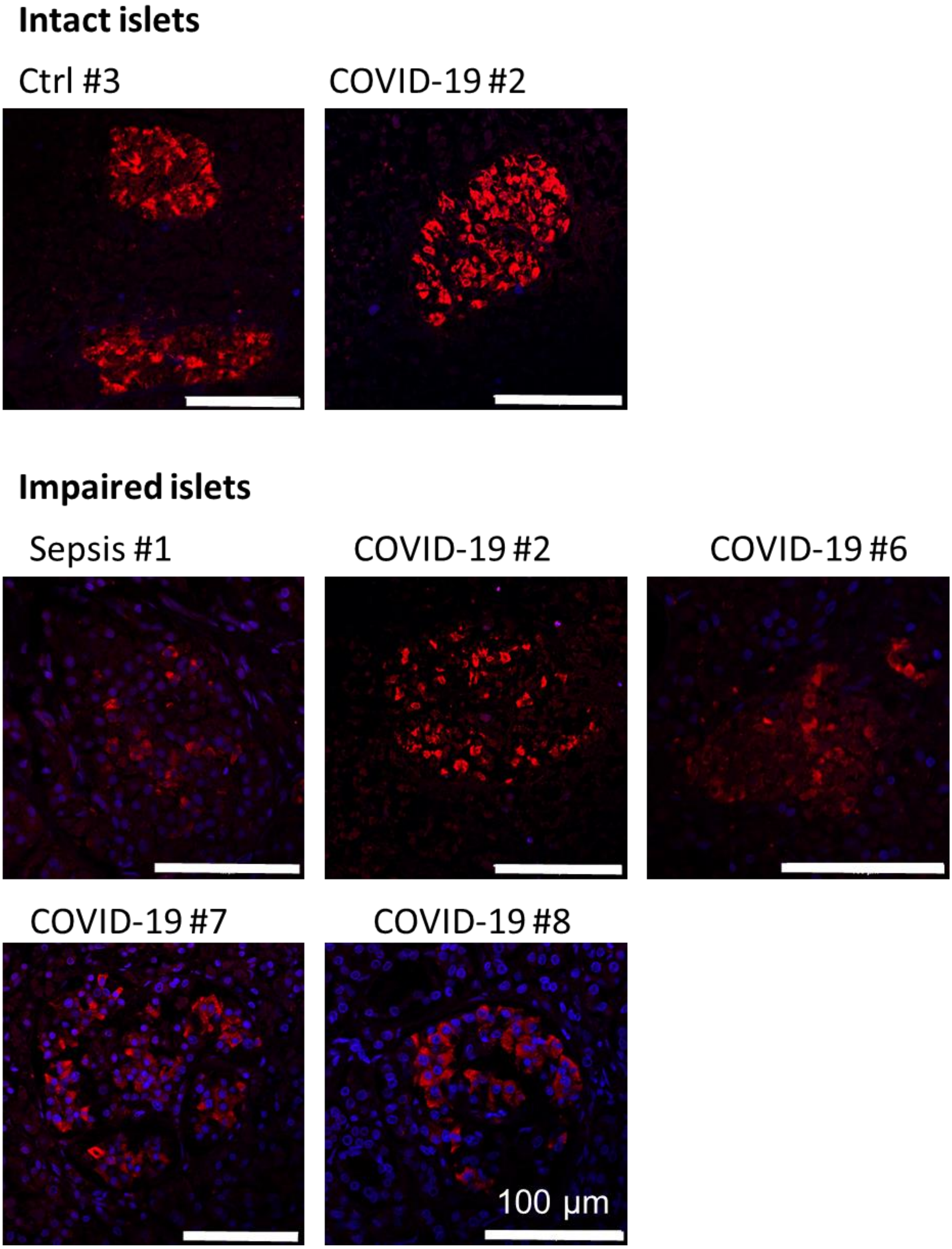

Supplementary Figure 2. Insulin stainings of selected pancreas control and COVID-19 patient tissues. In the control tissue, all islets are intact. In COVID-19 patients, intact and impaired islets are detected. Representative images from eight independent experiments (n=8) are shown.

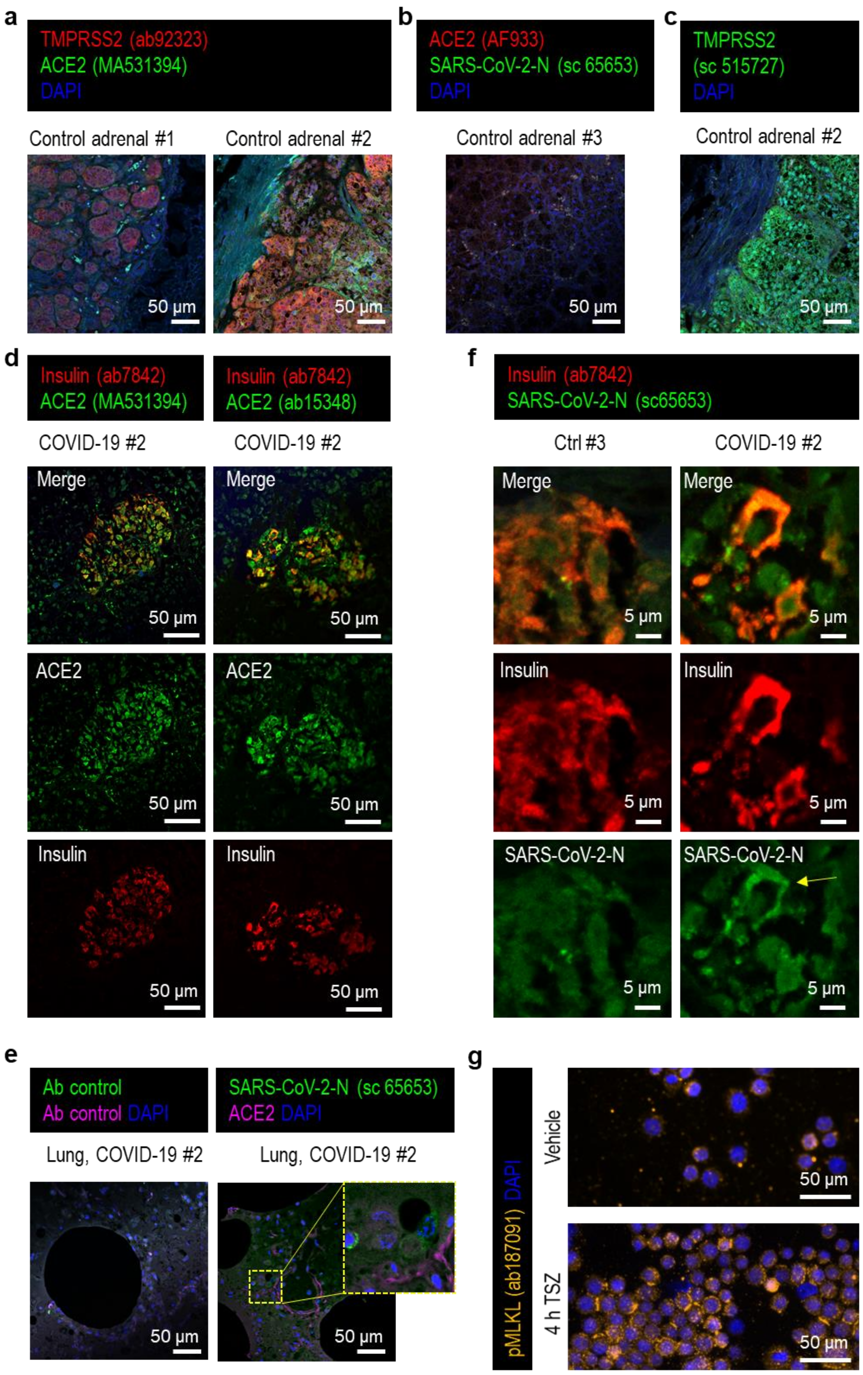

Supplementary Figure 3. Antibody validation. **a-c.** Different antibodies were tested in immunostainings on human adrenal tissue. Anti-TMPRSS2 (ab92323) worked and therefore this antibody was chosen for further stainings (n=3). Anti-TMPRSS2 (sc 515727) showed a similar staining pattern but also in the nucleus (n=1). Anti-ACE2 (MA531394) works (n=3). Anti-ACE2 (AF933) didn't work (n=1). No staining of SARS-CoV-2 Nucleocapsid protein (SARS-CoV-2-N) was observed on human control adrenal tissue (not from COVID-19 patients) (n=1). **d.** Two ACE2 antibodies (MA531394 and ab 15348) were compared on pancreas tissue and showed similar stainings (n=3). **e+f**. Anti-SARS-CoV-2-N (sc65653) works on positive lung control tissue and on pancreas tissue from the same patient (n=2). **g.** Anti-pMLKL (ab187091) was tested on HT29 cells where necroptosis was induced (n=2). Representative images are shown.

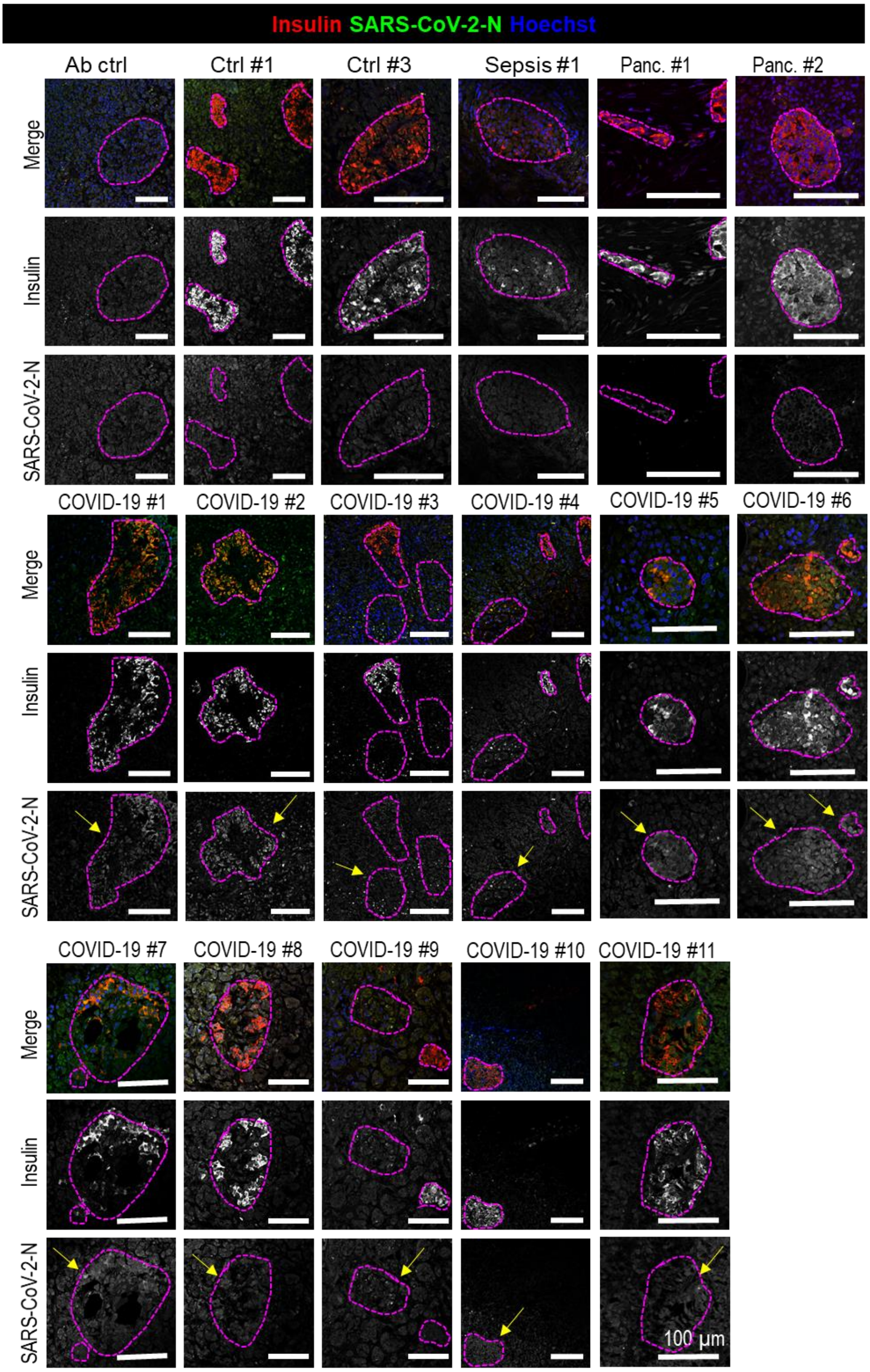

Supplementary Figure 4. SARS-CoV-2 infection leads to viral infiltration in the pancreas. Immunostainings of pancreas sections from COVID-19 patients using antibodies against insulin and SARS-CoV-2-N (sc65653). Secondary antibodies alone are seen in "Ab control". Islets are indicated. Representative images from three independent experiments (n=3) are shown. Scale bars, 100 µm.

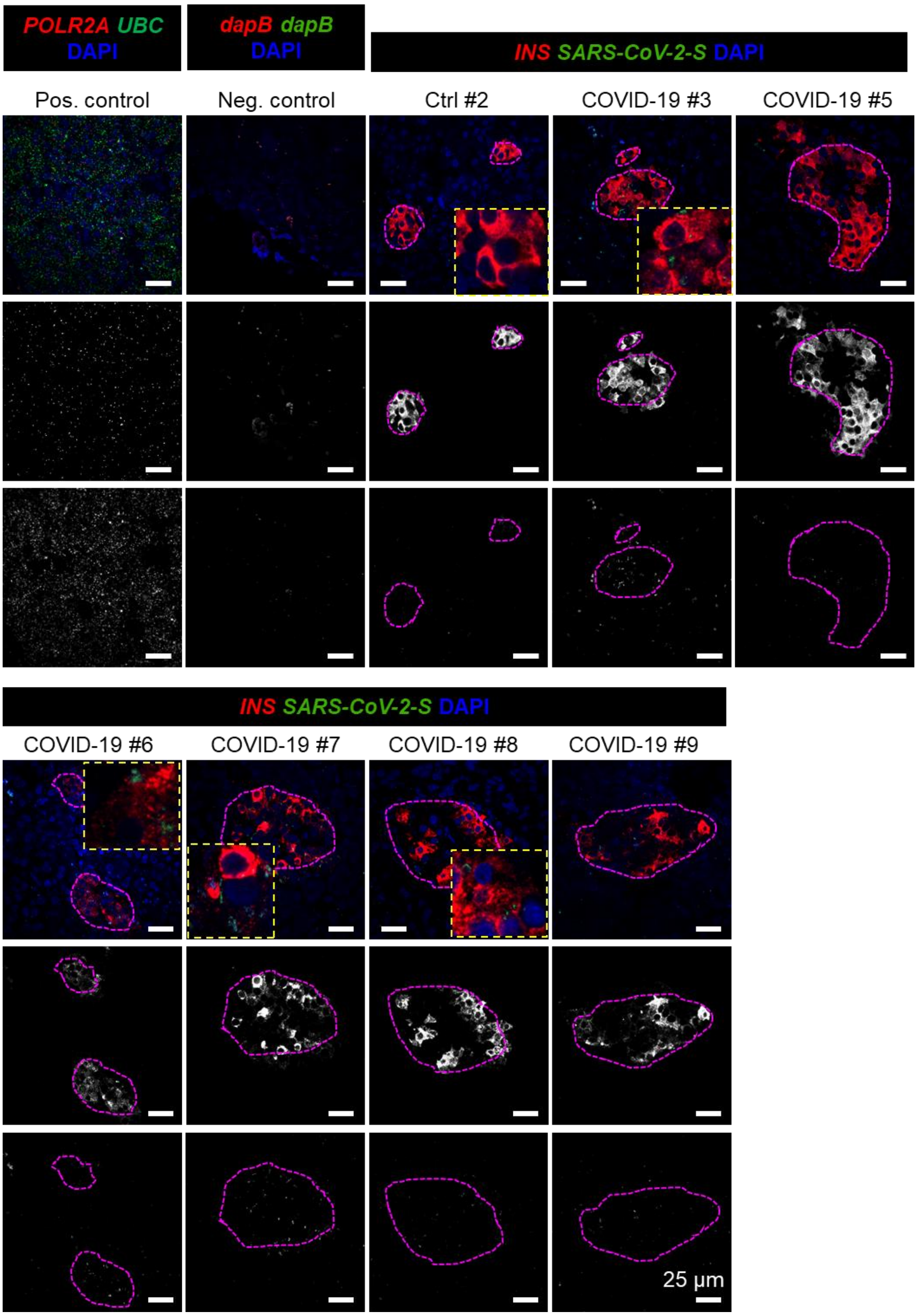

Supplementary Figure 5. SARS-CoV-2 infection leads to viral infiltration in the pancreas. RNA scope on pancreatic tissue from COVID-19 patients using probes targeting *INS* and *SARS-CoV-2-S*. As positive control, probes targeting *POLR2A* and *UBC* were used. As negative control, probes targeting the *DapB* gene from the Bacillus subtilis strain SMY were used. Islets are indicated. Representative images from two independent experiments (n=2) are shown. Scale bars, 25 µm.

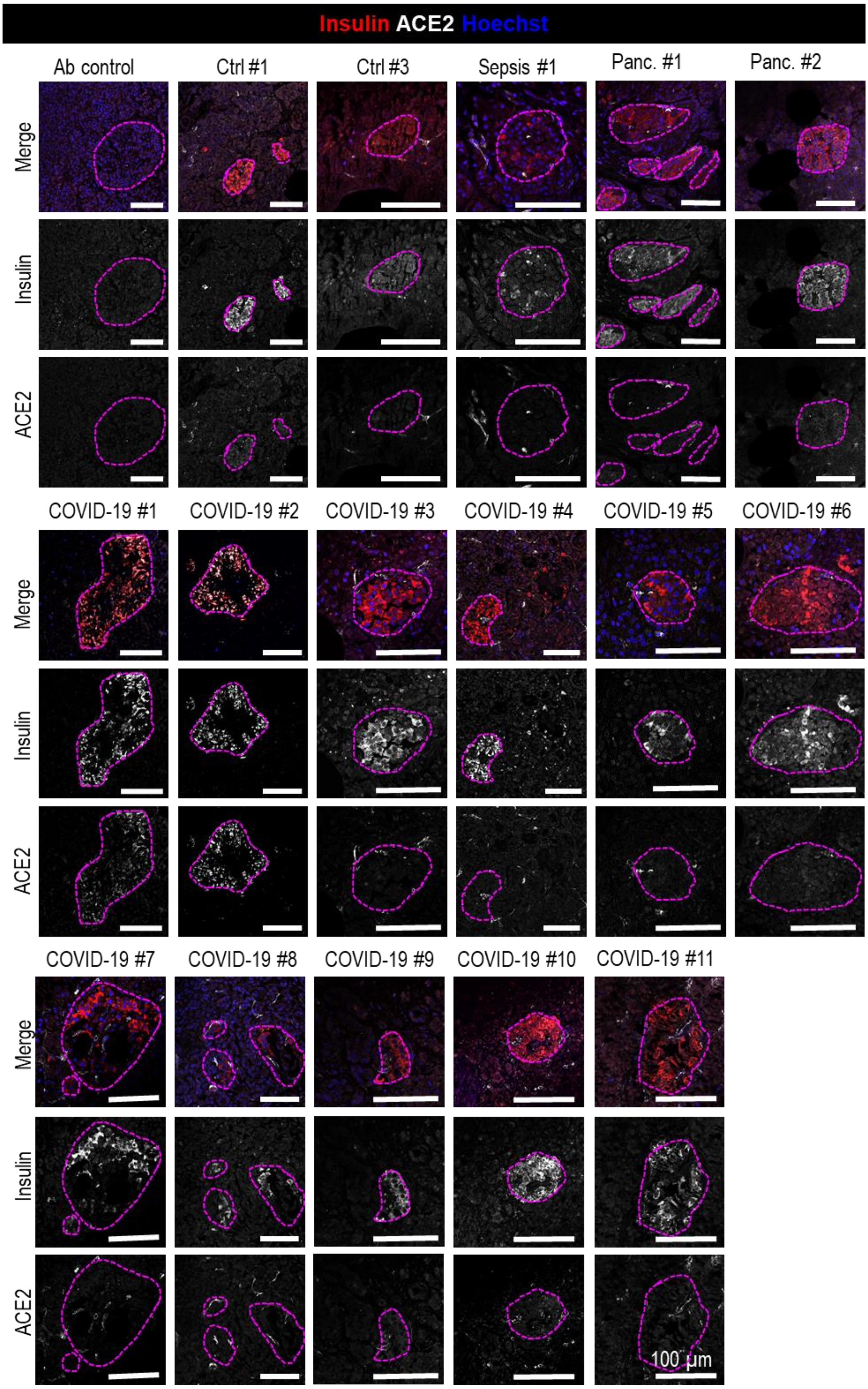

Supplementary Figure 6. The SARS-CoV-2 receptor ACE2 is expressed in the human pancreas. Pancreas sections from control and COVID-19 patients were stained for insulin to mark beta-cells. Additionally, double stainings for ACE2 were performed. Secondary antibodies alone are seen in “Ab control”. Islets are indicated. Representative images from three independent experiments)n=3) are shown. Scale bars, 100 µm.

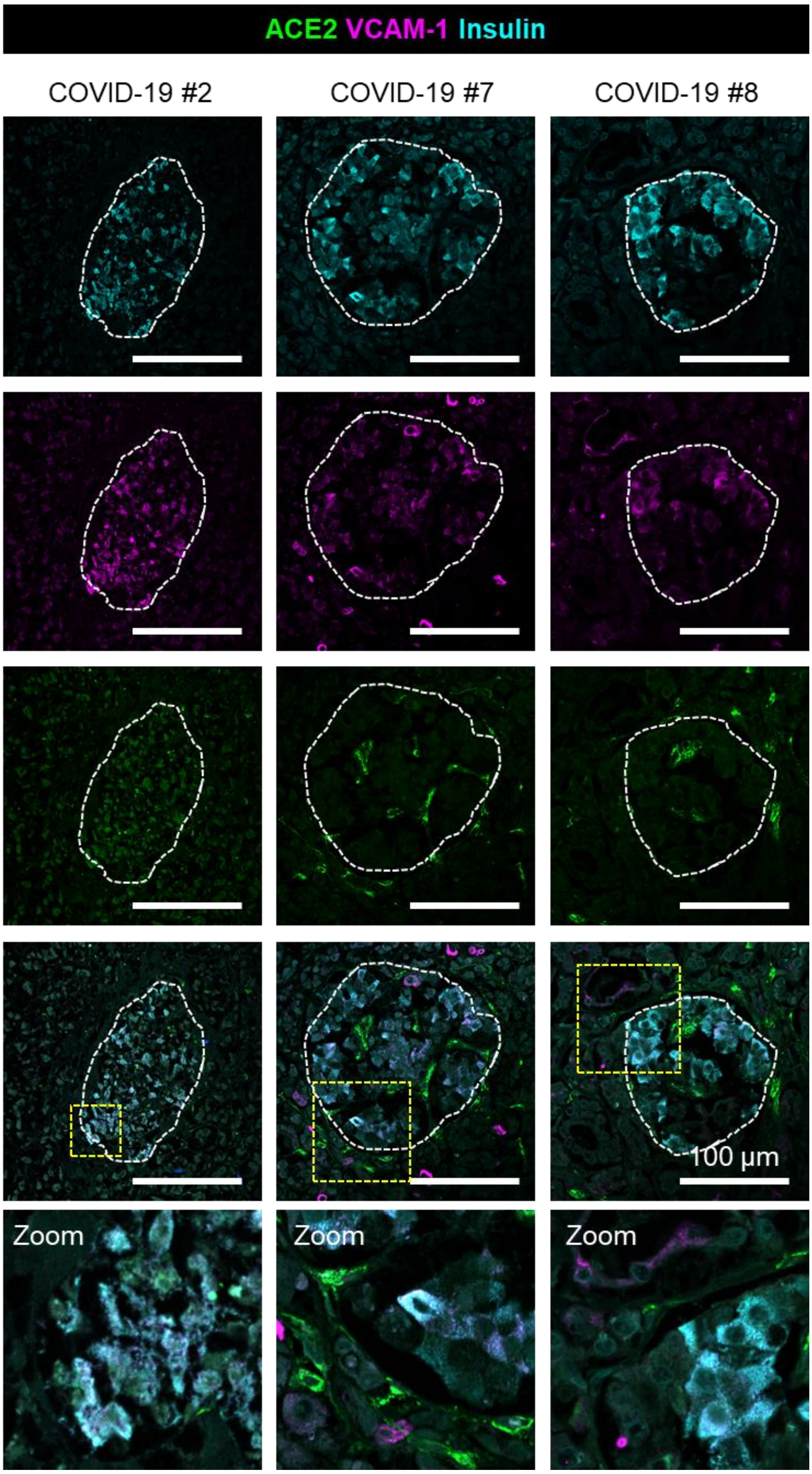

Supplementary Figure 7. Endothelial cells express ACE2. Immunostainings of pancreas sections from COVID-19 patients using antibodies against insulin, VCAM-1 and ACE2. Representative images from two independent experiments (n=2) are shown. Scale bars, 100 µm.

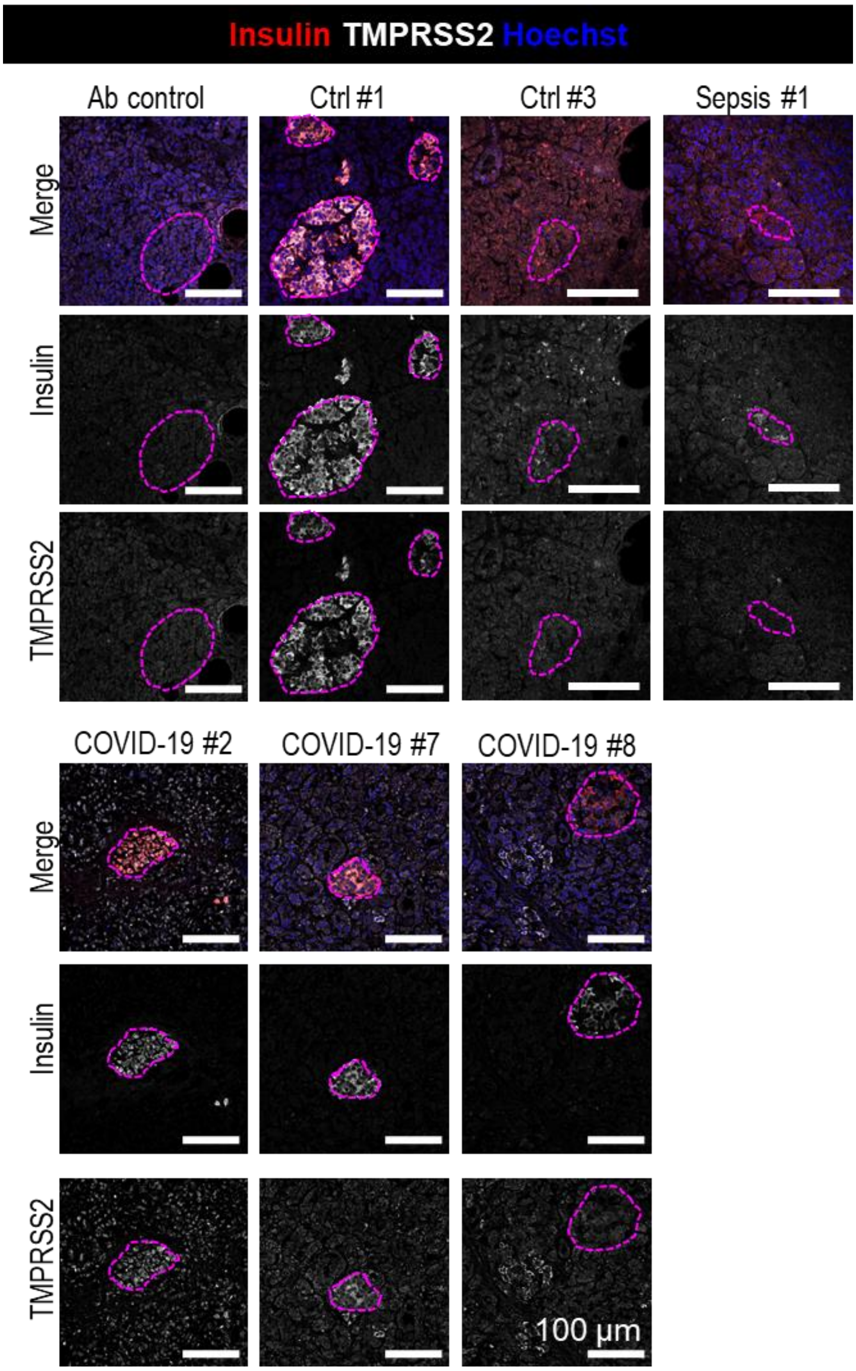

Supplementary Figure 8. SARS-CoV-2 protease important for cell entry is expressed in the human pancreas. Pancreas sections from control and COVID-19 patients were stained for insulin to mark beta-cells. Additionally, double stainings for TMPRSS2 were performed. Secondary antibodies alone are seen in “Ab control”. Islets are indicated. Representative images from three independent experiments (n=3) are shown. Scale bars, 100 µm.

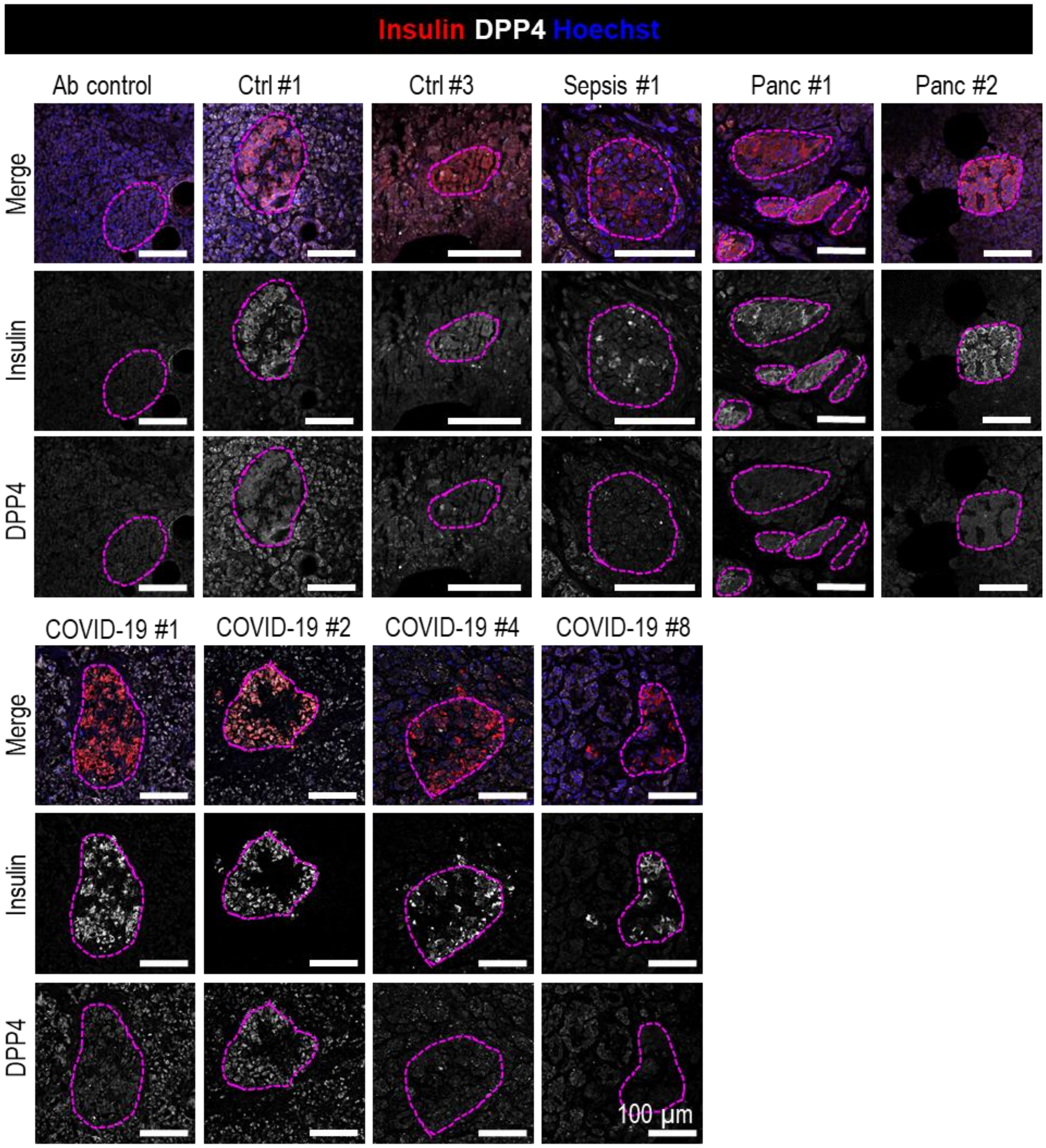

Supplementary Figure 9. SARS-CoV-2 alternative receptor DPP4 is expressed in the human pancreas. Pancreas sections from control and COVID-19 patients were stained for insulin to mark beta-cells. Additionally, double stainings for DPP4 were performed. Secondary antibodies alone are seen in "Ab control". Islets are indicated. Representative images from two independent experiments (n=2) are shown. Scale bars, 100 µm.

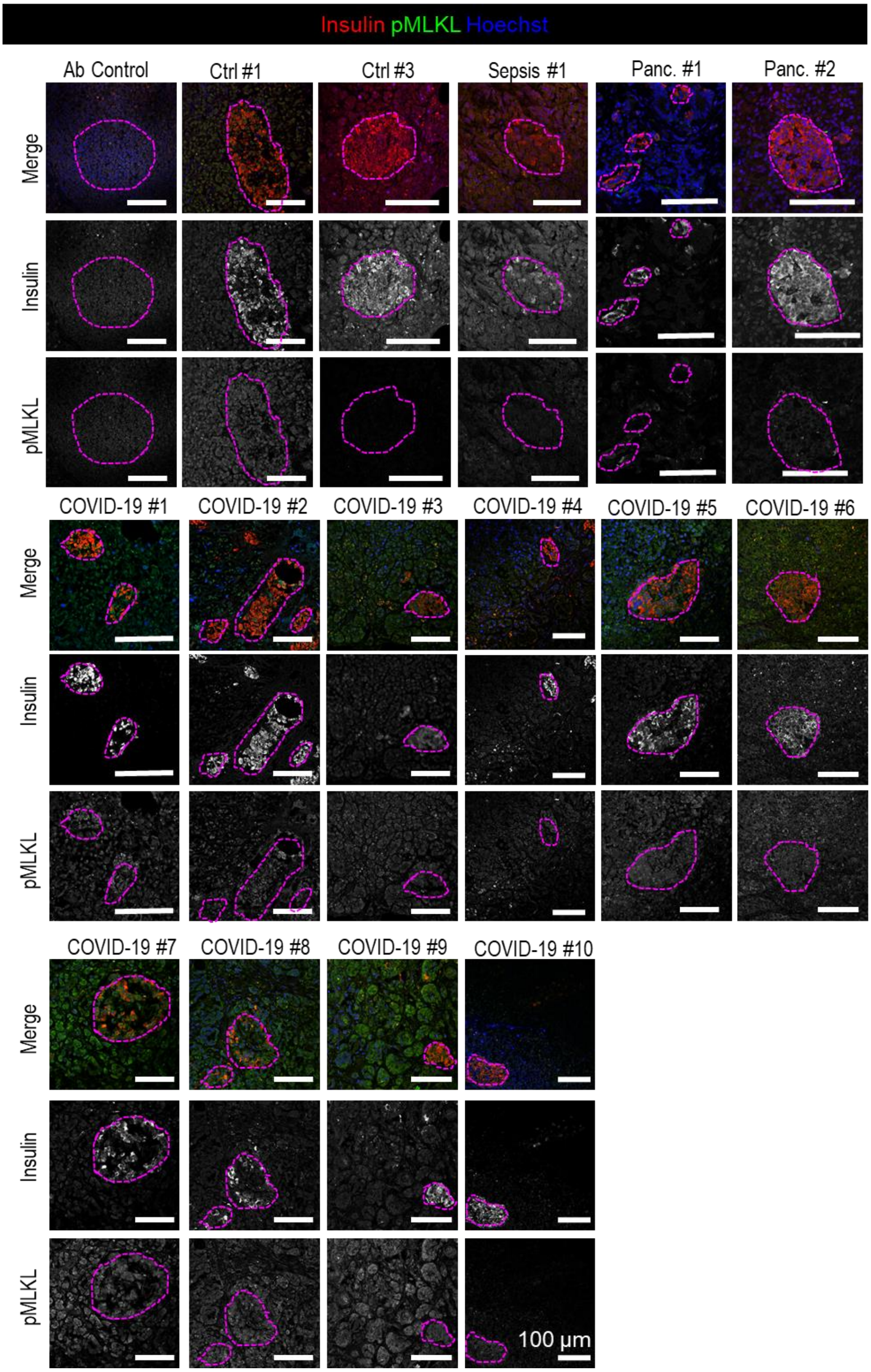

Supplementary Figure 10. SARS-CoV-2 infection leads to necroptosis in the pancreas. Immunofluorescent stainings of pancreas sections from COVID-19 patients using antibodies against insulin and pMLKL. Secondary antibodies alone are seen in "Ab control". Islets are indicated. Representative images from three independent experiments (n=3) are shown. Scale bars, 100 µm.

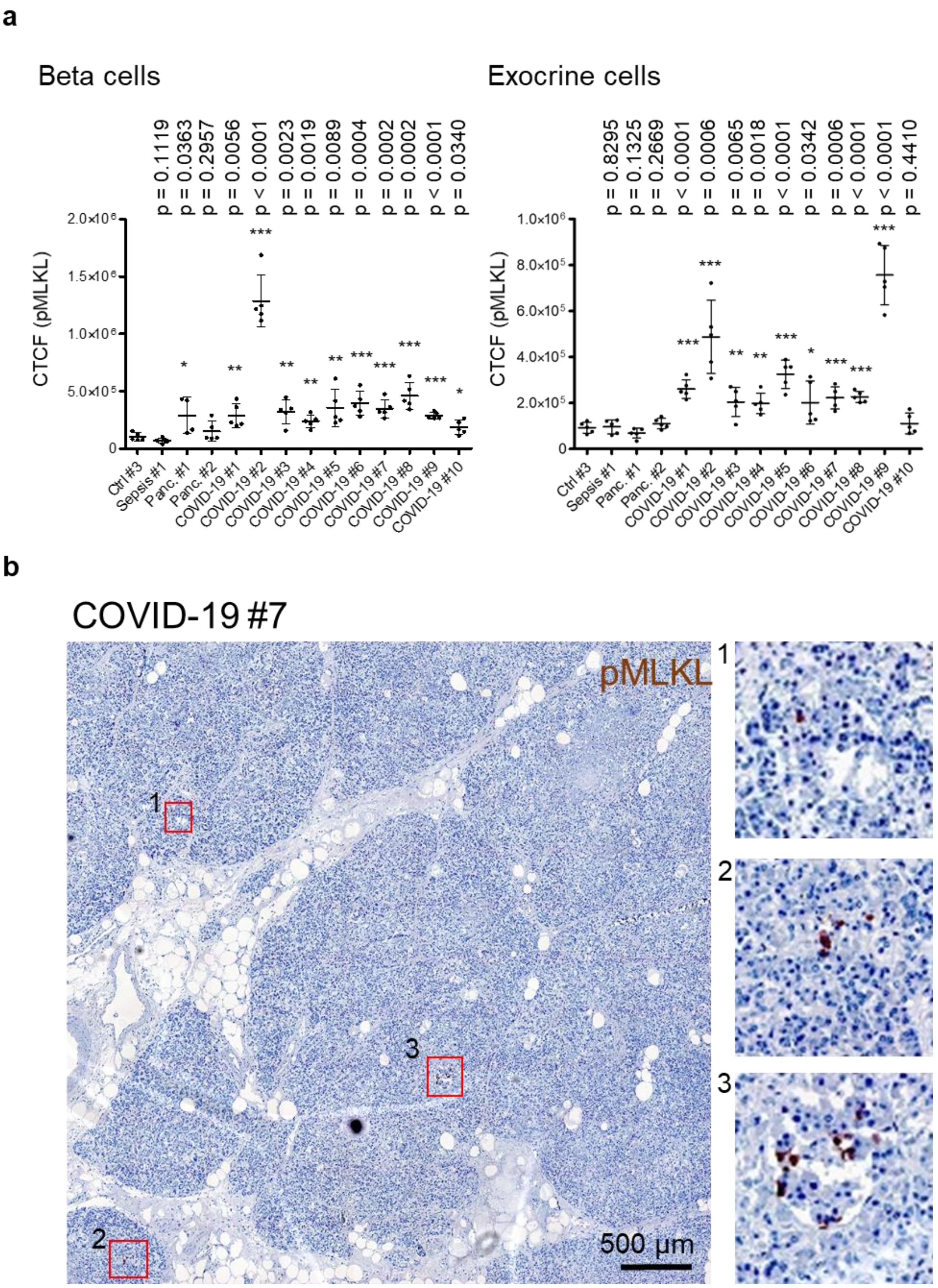

Supplementary Figure 11. SARS-CoV-2 infection leads to necroptosis in the pancreas. a Dot blots of pMLKL cellular fluorescence in beta-cells and exocrine cells measured on one (n=1) of three independent stainings in Supplementary Fig. 10. Quantification of calculated total cellular fluorescence (CTCF) was performed on n=5 cells from n≥2 images per patient. Data were analyzed by unpaired two-sided *t*-test by comparison with Ctrl #3. Data are presented as mean ± SD. p values are indicated. * p < 0.05, **p < 0.01; ***p < 0.001. **b** Immunohistochemistry of pMLKL in a pancreatic section from COVID-19 patient #7. A representative image from one experiment (n=1) is shown. Scale bar, 500 µm.

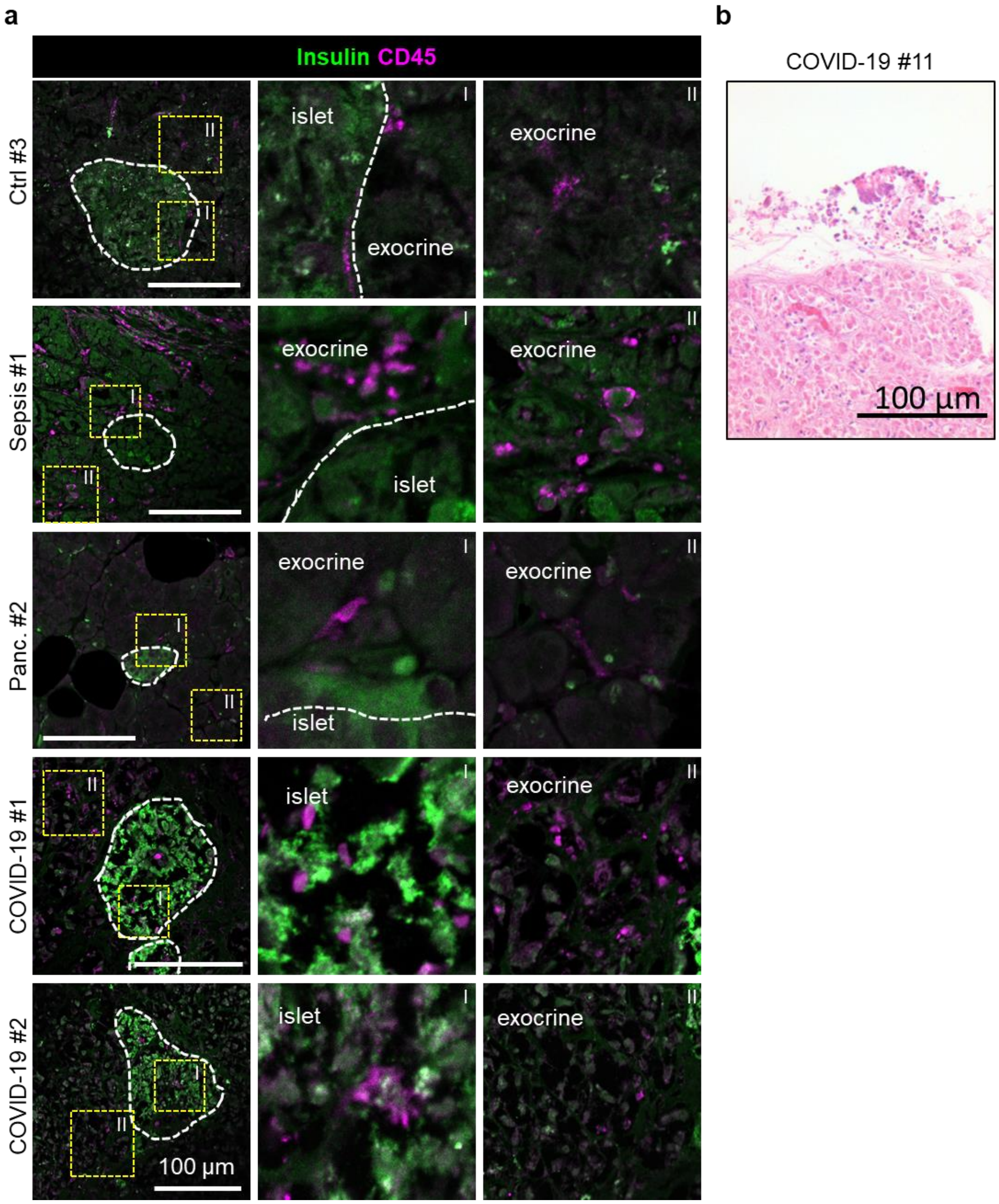

Supplementary Figure 12. SARS-CoV-2 infection leads to immune cell infiltration in the pancreas. a Immunostainings of pancreas sections from control and COVID-19 patients using antibodies against insulin and CD45. Islets are indicated. Representative images from two independent experiments (n=2) are shown. Scale bars, 100 µm. **b** H&E staining showing lymphocytic infiltration in tissue surrounding the pancreas. Representative image from one experiment (n=1) is shown. Scale bar, 100 µm.
