# Supplementary material for: Viral infiltration of pancreatic islets in patients with COVID-19
Source: Nat Commun. 2021 Jun 10;12:3534. doi: 10.1038/s41467-021-23886-3 (PMC8192507; doi:10.1038/s41467-021-23886-3)
Supplement: Supplementary file 2 — Peer Review File [file 41467_2021_23886_MOESM2_ESM.pdf]

REVIEWERS' COMMENTS

Reviewer #1 (Remarks to the Author):

Thanks for taking the time to address my comments

We would like to thank reviewer #1 for investing time on reviewing the manuscript again:

“Reviewer #1 (Remarks to the Author):

Thanks for taking the time to address my comments”
